# Revealing Spatial Patterns of Cultural Ecosystem Services in Four Agricultural Landscapes: A Case Study from Hangzhou, China

**DOI:** 10.3390/ijerph19159269

**Published:** 2022-07-28

**Authors:** Shan He, Chenxia Hu, Jianfeng Li, Jieyi Wu, Qian Xu, Lin Lin, Congmou Zhu, Yongjun Li, Mengmeng Zhou, Luyao Zhu

**Affiliations:** 1 College of Economics and Management, China Jiliang University, Hangzhou 310018, China; heshan33@cjlu.edu.cn (S.H.); ljfwinner@163.com (J.L.); s21071201031@cjlu.edu.cn (J.W.); xuqian@cjlu.edu.cn (Q.X.); 2 College of Humanities and Foreign Languages, China Jiliang University, Hangzhou 310018, China; linlin@cjlu.edu.cn; 3 Department of Land Resources Management, Zhejiang Gongshang University, Hangzhou 310018, China; congmouzhu@zjgsu.edu.cn; 4 College of Environment and Natural Resource, Zhejiang University, Hangzhou 310058, China; yongjunli@zju.edu.cn (Y.L.); luyao_zhu@zju.edu.cn (L.Z.); 5 School of Business, Changzhou University, Changzhou 213164, China; mm_zhou2022@163.com

**Keywords:** cultural ecosystem services, agricultural landscape, spatial patterns, societal preference

## Abstract

Monitoring and mapping agricultural cultural ecosystem services (CES) is essential, especially in areas with a sharp contradiction between agricultural land protection and urban development. Despite research assessing CES increasing exponentially in recent years, our knowledge of the CES of agricultural landscapes is still inadequate. This study used four types of agricultural landscapes in Hangzhou, China, as the study area, analyzed their CES spatial patterns, and explored their societal preferences by integrating the multi-sourced datasets, clustering algorithms, and Maxent model. The results indicated that hot spots of agricultural CES correspond to river valley plains, which were also easily vulnerable to urbanization. Moreover, we found that the CES level of paddy field and dry farmland were higher than tea garden and orchard. Based on the above spatial patterns of supply, demand, and flow of CES, we identified four groups of agricultural land by cluster analysis, distinguishing between significant, unimportant, little used, and potential CES. Further, our results showed that natural and human factors could explain societal preferences. This study can provide a valuable basis for stakeholders to develop balanced strategies by the aforementioned results.

## 1. Introduction

Cultural ecosystem service (CES) is a vital benefit contributed by ecosystems and generated by human–place relations [1,2]. In the Common International Classification of Ecosystem Services (CICES), CES is defined as “the nonmaterial and intangible benefits acquired from ecosystems” [3]. Due to their intangible and subjective characteristics, CES values are difficult to measure [1,4]. As researchers and practitioners have recognized the crucial role of CES in promoting human well-being and exceptionally motivated environmental action [5], a growing number of studies have started to address CES [1,2,6].

Agricultural land, a critical component of ecosystems that support human livelihoods, plays a role in producing food, fiber, and livestock fodder [7]. In addition to their primary functions, agricultural lands also have the potential to deliver CES, such as aesthetic appreciation, outdoor recreation, education, cultural heritage, etc. [8]. However, compared to green spaces, CES related to agricultural lands has received less attention.

China feeds 20% of the worldwide population with only 7% of global farmland, and there is a sharp contradiction between agricultural land protection and urban development [9]. Because the urbanization rate grew from 17.92% in 1978 to 83.60% by 2021 [10], the Chinese government has determined that it is urgent to protect agricultural land to maintain its productive functions. Additionally, the government put forward the “rural vitalization” strategy to address the contradiction of the uncoordinated urban–rural development, leading to the flourishing of rural tourism [11] and, subsequently, highlighting the corresponding agricultural CES [12]. However, flourishing rural tourism might pose a threat to the productive function of agricultural land. Thus, mapping and evaluating the agricultural CESs and then analyzing them for trade-offs or synergies with other services plays a crucial role in accurately identifying the direction of agricultural land use and, ultimately, maximizing the benefits of multiple ecosystem services of agricultural land [12]. That is also of paramount importance for the sustainable use and conservation of agricultural land [8].

Regarding the evaluation framework of CES, most research has generally focused on the supply (potential) of CES by survey-based or proxy-based methods [13,14]. In contrast, only a few scholars have considered demand in evaluating CES [2,15] using the economic valuation, participatory approaches, etc., to quantify CES demand level. Due to being time-consuming, the CES flow (actual use) regarding visit frequency (human preference) was usually carried out in local studies [16]. Due to the inaccessibility of datasets, little research has been performed on agricultural CES flow. Exploring the spatial patterns of CES supply, demand, and flow and revealing a comprehensive depiction of the performance of agricultural CESs can be more conducive to management, planning, and decision-making [1,12,17].

Comparing the differences in CES between agricultural land types is another issue to be addressed. As CES is determined by diverse human (infrastructure, etc.) and natural (topography, etc.) factors [1,18], their impact on CESs can vary with changes in land use types. Correspondingly, the supply, demand, and flow of CES could also be spatially heterogeneous across land-use types [1]. As most current studies only concentrate on one specific agricultural land type and assess CES across different agricultural landscapes or at large spatial scales [8,11], it is necessary and meaningful to conduct a more detailed agricultural CES study, comparing various agricultural land use types, such as paddy field, tea garden, and dry farmland. This would provide essential points of view for developing conservation plans and accurate land-use decision-making.

Social media datasets from internet platforms have been widely used to acquire data for mapping CES flow [6,12,17]. Since these data could reflect human preferences, they are geo-tagged, freely obtainable, and cover a wide range of areas [2]. These characteristics significantly contributed to CES research at large spatial scales. For instance, geo-tagged photographs acquired from social networks (Flickr, etc.) were used for evaluating CES flow by estimating visit frequency in Europe [2,17]. In China, the social media datasets, namely point of interest (POI) obtained from mobile maps or sharing platforms, also have excellent potential for CES analysis [12]. Moreover, their detailed tag information is incredibly beneficial for evaluating agricultural CES flow, including rural tourism, outdoor picking, fishing, etc. [19].

In summary, integrating supply, demand, and flow and comprehensively comparing their discrepancies among various land use types are large challenges in mapping and evaluating agricultural CES. This study took Hangzhou City in China as the study area and mainly contributed to three crucial aspects of agricultural CES. First, we drew on multi-sourced datasets to map agricultural CES. Among them, we evaluated supply based on spatially explicit indicators. The demand level was mapped by considering demographic information. Social media data were used as a proxy for CES flow. Second, a clustering analysis was conducted among CES supply, demand, and flow to reveal a comprehensive depiction of the performance of agricultural CESs. Third, we further explored the societal preferences with respect to agricultural CES, including quantifying agricultural POI distribution and the impacts of factors on preferences among diverse agricultural landscapes. The results of this study could provide support for sustainable management, planning, and decision-making of agricultural land.

## 2. Materials and Methods

### 2.1. Study Area

**Figure 1:**
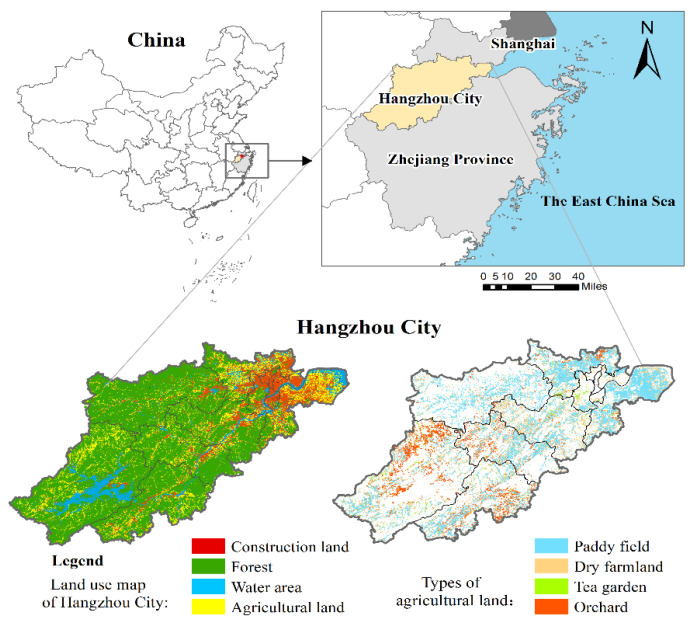
The location of the study area of Hangzhou.

As the capital of Zhejiang Province, China (latitude: 29°11′–30°33′ N, longitude: 118°21′–120°30′ E), Hangzhou is one of the most prosperous cities in southeast China, with a total area of 16,852 km^2^ (Figure 1). This city consists of 10 municipal districts, 2 counties, and 1 county-level city. The area of agricultural land (57% for paddy field, 15% for dry farmland, 13% for tea garden, and 15% for orchard) in Hangzhou was 2121 km^2^ in 2019, accounting for 12.59% of the total land area. Since the reform and opening up, Hangzhou has experienced rapid economic development, with GDP per capita rising from CNY 4614 in 1995 to CNY 15,1721 in 2021, ranking 16th out of 100 major cities in China. Moreover, the population growth rate of Hangzhou City is up to 3.57%/year, ranking 3rd in the country. These pose a severe challenge for protecting agricultural land.

Additionally, the stunning scenery and urban vibrancy of this city attract a large number of domestic (89.52 million in 2021) and foreign (0.18 million in 2021) tourists. Against the backdrop of China’s vigorous development of rural revitalization in 2017, rural tourism in Hangzhou has also been developing rapidly. In 2020, rural tourism in Hangzhou received 70.80 million visits, and its total operating income was CNY 6.93 billion. Hangzhou, thus, ranks first in the China Rural Tourism Development Index report, which makes the CES of agricultural land in Hangzhou a great concern.

Therefore, it is typical, representative, and necessary to study the cultural ecosystem services of agricultural land in Hangzhou City, where urban development and farmland conservation are in sharp conflict and rural tourism is developing rapidly.

### 2.2. Agricultural Social Media Datasets

We used the following categories of data obtained from different sources in the present study (Table 1). Among them, the social media data and agricultural POI within Hangzhou City were obtained from Dianping.com on 18 January 2022 to quantify the CES flow. Dianping.com is one of the most popular websites in China, with approximately 400 million users. Consumers can comment on the locations they have visited by tagging and uploading text, photographs, or videos. Not only does this data have precise location information, but it also reveals users’ preferences very well [20]. Considering the merits mentioned above, scholars usually use POI to map CES and conduct social or natural assessments [19,20].

Each POI has a series of labels, such as categories, longitudes and latitudes, rating value, popularity value, and the number of comments by users. The 750 most popular POIs (in terms of popularity value) with categories closely related to agricultural CES, including “picking garden”, “agritainment”, and “country club”, were used to analyze agricultural CES. To ensure the quality of the datasets, we further identified the land use types (paddy field, dry farmland, tea garden, or orchard) of each agricultural POI by spatially overlaying POIs with agricultural land polygons in ArcGIS software (ESRI Inc., Redlands, CA, USA). The POI was defined as the nearest agricultural land use type within 500 m of an agricultural polygon. Ultimately, we collected 692 valid agricultural POIs within the study area.

### 2.3. Methods

#### 2.3.1. Assessing Agricultural CES Supply

This study used the supply potential and accessibility of agricultural ecosystems dered to quantify the CES supply. Supply potential is defined as the opportunities of agricultural ecosystems to provide recreation or aesthetics to its beneficiaries, regardless of their use [1]. On the other hand, the services are provided when people visit these agricultural landscapes using traffic infrastructure, like roads, highways, railways, etc. [14].

We employed five indicators to map the supply potential of agricultural CES, namely shape, contiguity, landscape diversity, distance to water, and terrain ruggedness index (TRI). Previous studies have demonstrated that these indicators are closely related to CES supply [1,2,18]. Briefly, plots with regular shape and high contiguity could provide high visual views [12]; diverse landscape offers a high degree of recreational and visual appeal [17]; water body provides a variety of recreational opportunities and has a relatively high visual appeal in comparison with the surrounding area [24]; rough landscape (with its high TRI) offers many recreational opportunities and is visually more attractive than the flat landscape [1]. In this study, all datasets were converted to raster, normalized to values of 0–1, and resampled to a 90 × 90 m spatial resolution. The indicators above were considered equally contributing to CES supply potential; thus, they were overlaid to obtain the CES supply potential.

Accessibility of agricultural ecosystems determines whether CES can be used by society. Proximity to residential areas is critical for people using CES [1]. Therefore, we used travel time calculated by the cost distance tool in ArcGIS software to quantify the accessibility of agricultural ecosystems. Shorter travel time to a site promotes better accessibility of that site. Subsequently, the above supply potential was overlaid with an accessibility layer to obtain the agricultural CES supply map. See Table 2 for further details of the above indicators.

#### 2.3.2. Mapping Agricultural CES Demand

Demand represents the amount of the CES desired by society. In terms of demand mapping, there has not been a consensus yet [26]. Here, the spatial density of beneficiaries was used to express the CES demand level. Considering that agricultural CES was closely related to rural tourism, we defined the beneficiaries of agricultural CES as agritourists and residents [1]. To keep in line with supply, the two demographic indicators above were resampled to a 90 × 90 m spatial resolution and then added together to obtain the agricultural CES demand map.

#### 2.3.3. Quantifying Agricultural CES Flow

Flow refers to the actual usage of the CES by beneficiaries [12,17]. Two indicators, viewshed and popularity of agricultural POI, were integrated to quantify the CES flow. This study assumed that more viewshed overlaps obtained from agricultural POI promote more actual usage of agricultural CES by beneficiaries. The viewshed indicator was calculated in ArcGIS with agricultural POI and DEM data. Detailed parameter settings can be found in our previous publication [12]. In addition, the popularity indicator can reflect users’ preference distribution and visit frequency to agricultural landscape sites. It was estimated by the inverse distance weighting (IDW) method according to the popularity value of agricultural POI. Ultimately, the two indicators above were resampled to a 90 × 90 m spatial resolution and added together to obtain the agricultural CES flow.

#### 2.3.4. Exploring Societal Preferences for Agricultural CES

Societal preferences for agricultural landscape sites can be affected by landscape attributes such as topography, landscape diversity, transport infrastructure, etc. [27]. To explore societal preferences and explain the influencing mechanisms of landscape attributes on the spatial variations in CES flow within different agricultural land use types, we further conducted a relationship analysis between the popularity of agricultural POI and its corresponding landscape attributes. Regarding landscape attributes, we used the same six indicators in the CES supply above. Moreover, the Maxent (version 3.4.1) (Steven Phillips, MD, USA) [28] was used to conduct the relationship analysis. This method is commonly used to predict species distributions and has been demonstrated in previous CES studies [2,29]. Two sets of input data, occurrence localities of given observation points (agricultural POI with popularity values), and environmental variables (indicators in CES supply), were needed by the Maxent model. Ultimately, it can output the effects of indicators in preference distribution, including the relative contribution and importance as well as the response curve of each variable.

#### 2.3.5. Statistical Analysis

To ensure consistency, the CES supply, demand, and flow values above were all rescaled from 0 (low) to 1 (high). Moreover, we classified all values into three levels (high, medium, and low) by the geometrical interval classification method. To visualize the spatial pattern of the above three datasets better, we further conducted the Getis-Ord Gi* statistical analysis, which can identify hot and cold spots of the CES supply, demand, and flow. In addition, a k-means cluster analysis was carried out to obtain CES groups with similar patterns of supply, demand, and flow. We also counted the distribution of CES values and CES groups for different agricultural land types. In the present study, all spatial analysis was completed in ArcGIS 10.2 (ESRI Inc., Redlands, CA, USA).

## 3. Results

### 3.1. Patterns of Agricultural CES Supply, Demand, and Flow

**Figure 2:**
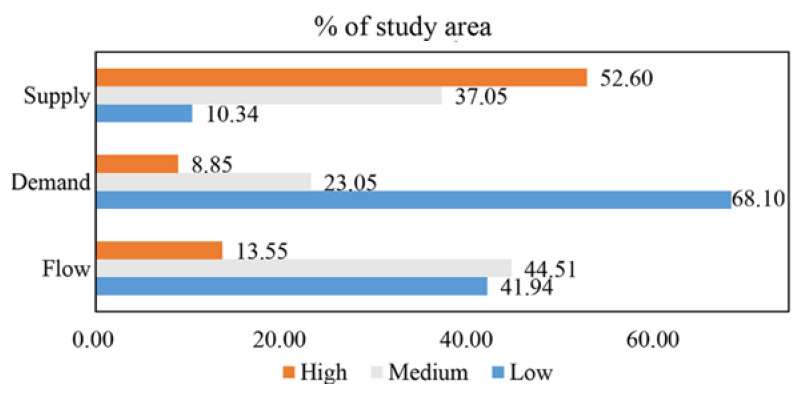
Distribution of agricultural CES values.

As presented in Figure 2, agricultural land with different CES supply, demand, and flow levels was distributed unequally within the study area. Most agricultural land (52.60%) showed a high CES supply value, whereas it was low in 10.34% of agricultural land. In terms of CES demand, it was high for only 8.85% and low for 68.10% of the study area. High CES flow was characteristic of 13.55% of the agricultural land, while medium and low values were equally distributed.

**Figure 3:**
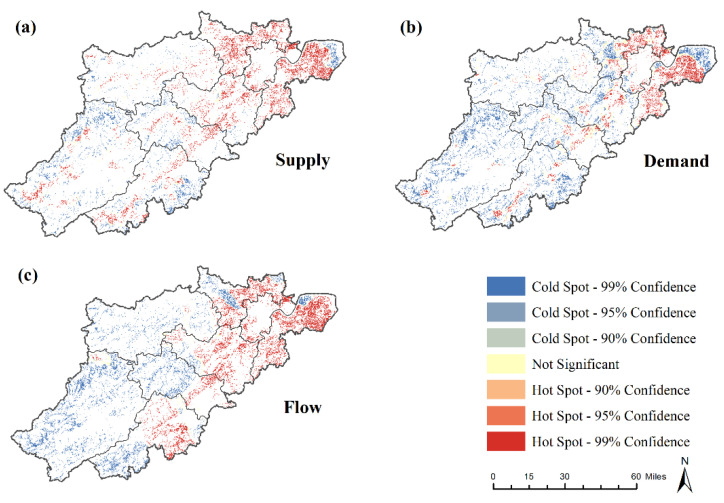
Patterns of hot and cold spots of (**a**) supply, (**b**) demand, and (**c**) flow maps.

With respect to spatial patterns, the agricultural CES supply, demand, and flow were spatially clustered in different locations within the study area with a diverse spatial pattern (Figure 3). Among them, high supply was observed in the flat areas and along the waterways, and the low supply was primarily scattered at the edge of the study area. High demand was located around densely populated areas, while the remote mountainous regions provided low demand. Compared with supply and demand, the distribution of flow was more clustered. The high flow was concentrated in the suburbs, and areas far from the downtown were characterized by low agricultural CES flow.

From the perspective of different agricultural land use types, overall, the CES level of paddy field and dry farmland was relatively high compared with the tea garden and orchard (Table 3). Specifically, 6.53% of dry farmland had high demand, whereas it was high in 5.82% of paddy field. Contrary to demand, the paddy field (64.85% and 17.97%) showed higher CES supply and flow than dry farmland (42.57% and 12.45%). They had comparable CES supply capacity and demand levels regarding tea garden and orchard. In terms of flow, a high level was characteristic in 8.36% of tea garden, while only 3.64% of the orchard had high flow.

### 3.2. Integrated Analysis of Agricultural CES

**Figure 4:**
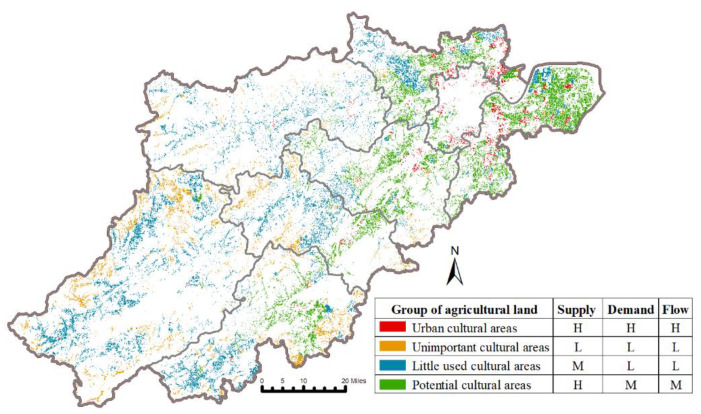
Groups of agricultural land with similar CES supply, demand, and flow values, produced by cluster analysis.

Based on the analysis above, we further identified four agricultural land groups differing in supply, demand, and flow levels to enhance the comprehensive understanding of agricultural CES (Figure 4). The ‘urban cultural areas’ group accounted for 5.14% of the total agricultural land and was characterized by generally high CES values. It was concentrated around the downtown area. Additionally, 40.43% of the study area, mainly located in the suburbs, was in the ‘potential cultural areas’ group, representing high supply and medium demand and flow. The ‘little used cultural areas’ group constituted 38.80% of the study area. This group was characterized by medium supply, low demand, and flow. These agricultural lands were often located far from the urban areas. The ‘unimportant cultural areas’ group indicated low CES values; it included 15.63% of the total agricultural land in remote mountainous areas with poor infrastructure.

**Figure 5:**
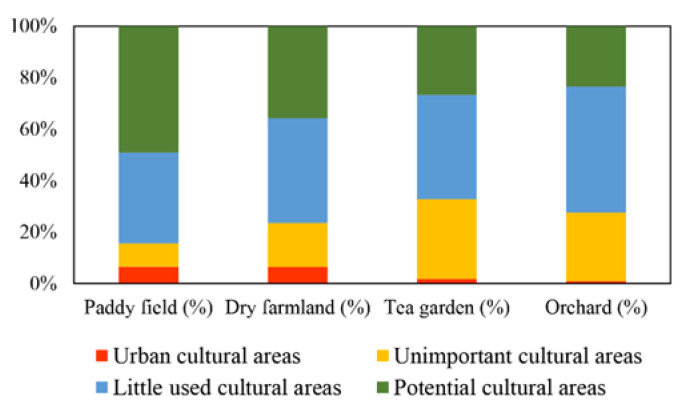
Distribution of different CES groups in each agricultural land use type.

The distribution of CES groups in each agricultural land use type is presented in Figure 5. The proportion of ‘urban cultural areas’ and ‘potential cultural areas’ in paddy field was the highest of all agricultural land use types, followed by dry farmland. ‘Little used cultural areas’ were evenly distributed across all land use types. The ‘unimportant cultural areas’ were mainly distributed in the tea garden and orchard.

### 3.3. Societal Preferences for Agricultural CES

**Figure 6:**
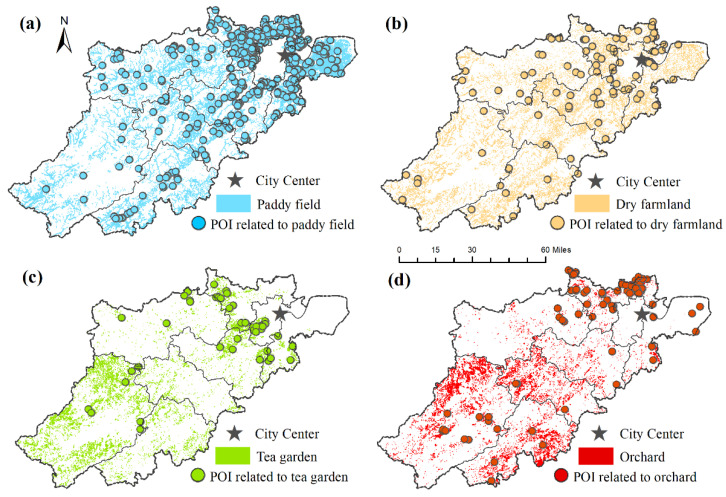
Distribution of different agricultural land types and the location of POIs related to each of them: (**a**) paddy field, (**b**) dry farmland, (**c**) tea garden, and (**d**) orchard.

Agricultural POI’s quantity and pattern reflected an uneven distribution across diverse agricultural land use types (Figure 6). POI-related tea garden and orchard accounted for 7.23% and 12.43%, respectively. They were mainly concentrated in proximity to the city center, opposite the patterns of tea garden and orchard (situated primarily in western and southern areas away from the city). Compared with POI-related tea garden and orchard, the distribution of POI-related paddy field (65.90%) and dry farmland (14.45%) was more scattered. In addition to the urban areas, it was also found in remote rural areas.

As exhibited in Table 4, accessibility was paramount among the various indicators affecting CES flow in all agricultural land use types. CES flow of paddy field and tea garden were closely related to TRI. Contiguity and landscape diversity contributed significantly to CES flow in dry farmland and orchard. Additionally, distance to water and the shape of the land are essential factors to CES flow in the orchard and paddy fields.

**Figure 7:**
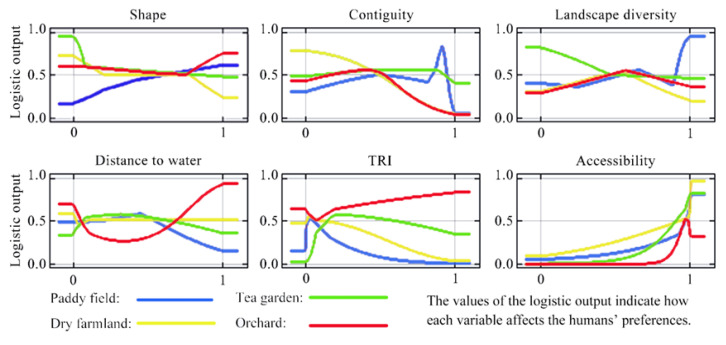
Response curves, produced by the Maxent model, of each environmental indicator affecting CES flow patterns.

The response curves indicated the mechanisms of each environmental indicator affecting CES flow patterns (Figure 7). Obviously, the impact mechanisms of the indicators were diverse. For instance, the CES flow of orchard was strongly correlated with undulating terrain and convenient transportation. High contiguity, near water bodies, and convenient transportation greatly impacted the flow of the tea garden. The flat terrain and convenient transportation made the CES of paddy field and dry farmland more accessible for human use. It is worth noting that, generally, regular shape, higher contiguity, and landscape diversity greatly favored the usage of CES in paddy fields, while this trend was opposite in dry farmland.

## 4. Discussion

Evaluation of CES remains challenging due to its extreme dependence on human perception and its disconnection from ecological processes [4]. In the present study, we addressed the challenges of mapping and analyzing spatial patterns of supply, demand, and flow across different agricultural landscape societies, providing vital information for decision-making and land-use policies.

### 4.1. Mapping Agricultural CES

Unlike other ecosystem services, CES arises from nature perception rather than from nature [30]. The supply of CES only represents the potential and opportunity of an ecosystem to provide such services [1]. In this study, we analyzed the supply capacity of agricultural CES by considering both natural and human factors. Some previous studies reported that outdoor recreation was provided mainly by mountain areas [1,31]. In contrast, our results revealed that supply hot spots correspond to most river valley plains within the study area. These regions have also become priority areas for urban development due to their superior natural advantages, such as flat terrain and proximity to water bodies, etc. It also underlines the specificity of the agricultural landscape, whose usage and conservation are in sharp conflict. Therefore, our findings highlighted the importance of analyzing agricultural CESs. It is necessary to analyze agricultural CES’s spatial patterns to inform precise and sustainable use options for agricultural land.

Questionnaires and interviews were frequently used in previous studies to quantify CES demand [18,32]. These methods were direct but time-consuming, costly, and challenging to present spatial information [30]. This study mapped the agricultural CES demand level based on geographic demographic data (beneficiary distribution) at high spatial resolution, including residents and rural visits. Compared with the traditional method, this method is efficient and can cover different areas of the study area [1]. Additionally, detailed spatial information allows CES to be linked to other ecosystem services. In this study, areas with high demand correspond to large urban areas with a dense population, which is consistent with the results of previous studies.

Flow refers to the actual use of CES, which is intangible and challenging to quantify [17]. In recent years, geo-tagged photos uploaded by users on social media (Flickr, etc.) have been used to map CES flow [1]. However, the platforms above are not popular in many countries. This study used the agricultural POI freely acquired from social media, namely Dianping.com, to analyze the agricultural CES flow. The popularity value of POI reflects the preference distribution and visit frequency of users to agricultural landscapes. To take aesthetics into account, we added the viewshed value to the popularity value to quantify the agricultural CES flow. This method has the merits of the previous approach (geo-tagged photos, etc.) and can also be more specific to the agricultural landscape. Similar to previous studies [1,33], hot flow spots were concentrated in downtown and suburban areas near cities in this study, indicating that humans prefer to use agricultural CESs provided by nearby destinations.

In previous evaluations on CES, researchers usually focused on the difference in CES in different land use types, such as urban green, arable land, forests, wetlands, etc. [1,2,29]. In the meantime, another study demonstrated CES variations between the urban center and suburban areas [6]. However, there is little in-depth discussion on the difference in spatial patterns of CES among diverse agricultural land use types. In addition to farmland, agricultural land, such as tea garden and orchard, are also part of the countryside and carry a variety of benefits for farmers, including economic, social, ecological, and recreational purposes [34]. While rapidly developing rural tourism brings economic benefits to the countryside, it also somehow threatens the productive and ecological benefits of the agricultural landscape [35]. Therefore, in-depth exploration of the CES for different agricultural land types can help elaborate land use planning and conservation programs. In this study, we have compared and analyzed the CES of four agricultural landscapes, including paddy field, dry farmland, tea garden, and orchard. The result showed that the CES level of paddy field and dry farmland were higher than that of tea garden and orchard, which is related to the site condition of different agricultural land use types. In the study area, most of the agricultural land is located near rural dwellings, thus making it easier for the farmers to cultivate and manage it on a daily basis. On the other hand, tea garden and orchard are located at a relatively high geographical altitude, which is not conducive to the performance of their CES.

### 4.2. Integrated Analysis of Agricultural CES Supply, Demand, and Flow

Based on the above spatial patterns of agricultural CES supply, demand, and flow, we further identified four agricultural land groups to enhance understanding of agricultural CES and provide suitable information for management and planning. We found that agricultural landscapes in proximity to urban areas belonging to ‘urban cultural areas’ have high CES values [1]. The data is precise because of the unique characteristics of the agricultural landscape, as we discussed earlier. High-quality agricultural land is often located near residential properties, easily accessible, and close to water sources [36], making it in high demand and use and high supply. These agricultural land resources are under pressure due to growing population and land transformations [37]. Thus, policymakers should figure out whether the agricultural CES supply within this group would be excessive. Moreover, attention also needs to be paid to the ecological and land transformation issues arising from the transitional exploitation of these agricultural lands [12].

Most of the agricultural land located in the suburbs belongs to the ‘potential cultural areas,’ as these areas offer high natural potential but may lack the necessary infrastructure [1]. They have become a reserve for city dwellers to experience the countryside and enjoy recreation. The municipalities of ‘little used cultural areas’ group in mountain areas might enhance the supply capacity by promoting the natural attributes of agricultural land and creating the rural tourism infrastructure (traffic, accommodations, parking lots, public toilets, etc.), which meets the psychological and activity demand of rural tourists [1]. Additionally, combining farming culture and agricultural land may also be an opportunity to enhance these landscapes’ attraction to visitors. Regarding the agricultural land within the ‘unimportant cultural areas’ group, strengthening the ecological environment management and production capacity may be the key to their sustainable development [12].

As demonstrated in previous studies, overuse of CES may lead to various social and environmental problems, like noisiness, crowdedness, and air pollution [35,36]. Excess supply, in turn, leads to wasted resources and threatens other services in the agricultural landscapes [12]. Furthermore, balancing the supply potential and infrastructure in rural tourism is crucial in influencing agricultural CES flow [1,2]. Hence, it is necessary to integrally analyze the agricultural CES supply, demand, and flow to contribute to the balanced strategies above.

### 4.3. Using Social Media to Assess Societal Preferences for Agricultural CES

To achieve more detailed information on societal preferences and thus inform future management and sustainable use of agricultural resources, this study has developed further assessment of the top agricultural POI in terms of popularity value. The variations in distribution and quantity of POI across diverse agricultural land use types can be connected to several factors. The high density of POI in paddy field and dry farmland may be related to proximity to residential areas and convenient transportation [33]. Lower CES values may influence the lower density of POI in tea garden and orchard [1]. In contrast to other studies [1,31], people prefer relatively flat landscapes with convenient transportation for agricultural recreation such as vegetable picking, farming experience, and angling in this study. Thus, the paddy field and dry farmland in river valley plains showed higher CES flow than in the tea garden and orchard.

Moreover, we found a consistent spatial pattern of farmland and POI related to farmland (paddy field and dry farmland). They are mainly concentrated near the downtown areas and were also found in remote rural areas [12]. Whereas the orchards (tea garden and fruit orchard) were mainly located away from the city center, the POIs related to orchard were concentrated near the urban areas. People prefer to pick vegetables and fruits grown on farmland (strawberries and blueberries, etc.) instead of picking tea and fruits grown on orchards (citrus and peach, etc.). The underlying reason is that people prefer to select destinations close to them to conduct agricultural recreation [1,12].

In addition to spatial analysis, this study also analyzed the factors influencing human preferences. We found that the impact mechanisms of the indicators were diverse [1,2]. Among them, accessibility played a crucial role in societal preference for agricultural CES [1,12], which is consistent with previous studies that conducive infrastructure conditions facilitate more recreational activities [31,33]. Furthermore, we found a negative relationship between agricultural POI density and TRI, which can be explained by lower accessibility to agricultural landscape characterized by undulating terrain and low human interference within the study area [1]. Similar to other studies [1,38], we also found that high contiguity and nearness to water bodies had significant impacts on the flow of the tea garden, emphasizing the importance of landscape views for aesthetic appreciation activities. Our study also indicated that people prefer to visit tea gardens for aesthetic appreciation rather than for recreational activities.

### 4.4. Limitations

Some considerations still need to be addressed. In terms of the datasets, the residents and agritourists were summed to present the CES demand. Among them, the agritourists at the county level might lead to the loss of some detailed information. In addition to the resident in the pixel, all residents in the entire county where the pixel is in or the neighboring county also can be considered as demand. Therefore, higher-resolution demographic data generated from mobile phone positioning will contribute to mapping the demand in the future study. Regarding the method, Maxent was used to evaluate the societal preferences for agricultural CES in this study. As this model is commonly used to predict species niches and distributions with specific assumptions, it is desirable to confirm whether it is the best method to evaluate the societal preferences for CES further. In our future study, we will include more accurate methods to explore societal preferences for agricultural CES.

## 5. Conclusions

China is experiencing rapid development in agritourism. Therefore, evaluating agricultural CES is essential and urgent, especially in areas with a sharp contradiction between agricultural land protection and urban development. However, compared with green spaces, CES related to agricultural lands has been given less attention. Moreover, integrating supply, demand, and flow and comparing their discrepancies among various land use types are the significant challenges of mapping and evaluating agricultural CES. Thus, this study addressed these challenges by integrating multi-sourced data and the Maxent method.

In terms of mapping the spatial patterns of agricultural CES, both natural and human factors were conducted to analyze the supply. At the same time, geographic demographic data were used to map the demand. Moreover, the agricultural POI freely acquired from social media was selected to quantify the flow. Our results showed that significant demand areas correspond to urban areas, and hot spots of flow were concentrated in suburban areas. Contrary to previous studies, the high supply was provided mainly by valley plains. These areas had also become priority areas for urban development, underlining the specificity of the agricultural landscape and highlighting the importance of analyzing CES to inform precise and sustainable use options for agricultural land.

Based on the spatial patterns of supply, demand, and flow of agricultural CES above, the study area was divided into four agricultural land groups by the k-means clustering algorithm. As described above, the grouping of agricultural land could contribute to balanced strategies between supply, demand, and flow, then further favor the development of specific policies. In this study, we also exerted CES variations of diverse agricultural land use types, including paddy field, dry farmland, tea garden, and orchard. Our results showed that the CES level of paddy field and dry farmland were higher than tea garden and orchard, related to the site condition of different agricultural landscapes.

Additionally, this study further assessed the societal preferences for agricultural CES using social media. For the POI density, the high density of POI in paddy field and dry farmland may be related to proximity to residential areas and convenient transportation. Lower CES values may influence the low density of POI in tea garden and orchard. Our results showed that accessibility played a crucial role in societal preference for agricultural CES regarding the impact mechanisms of indicators. Additionally, people generally prefer flat destinations to conduct agricultural recreation. Generally, our results can provide a valuable basis for further research and relevant input to inform stakeholders to develop balanced strategies.

## Figures and Tables

**Table 1:** Description of the datasets utilized in this study.

Data	Type	Resolution	Source
Agricultural POI	Shapefile	750 points	Open platform of Dainping.com website
Land use survey data ^a^	Shapefile	1:10,000	Land and Resources Bureau of Hangzhou
Road network	Shapefile	1:10,000	Open street map [21]
Digital elevation model	Geo Tiff	30 m	Geospatial data cloud [22]
Agritourists	Geo Tiff	County	Hangzhou Statistical Yearbooks [10]
Residents	Geo Tiff	100 m	WorldPop project [23]

^a^ The land use survey data includes agricultural land (paddy field, dry farmland, tea garden, and orchard), water body, residential land, and other land use types.

**Table 2:** Description of the indicators utilized in assessing agricultural CES supply.

Indicator	Connotation	Method
Shape	Complexity of shapes of agricultural land patches	Calculated based on the perimeter and area of the agricultural land patches [25]
Contiguity	Degree of contiguity of agricultural land patches	
Landscape diversity	Landscape diversity index	Number of land use types per km^2^ [18]
Distance to water (m)	Distance to the nearest water bodies	Euclidean distance [2]
TRI	Terrain Ruggedness Index	TRI classes [1]
Accessibility	Travel time from residentials to agricultural land	Cost distance [1]

**Table 3:** Distribution of agricultural CES values for different agricultural land use types.

Types of Agricultural Land	Supply (%)			Demand (%)			Flow (%)		
	H	M	L	H	M	L	H	M	L
Paddy field	64.85	30.98	4.17	5.82	14.91	79.27	17.97	49.07	32.96
Dry farmland	42.57	43.49	13.93	6.53	10.13	83.33	12.45	43.61	43.95
Tea garden	37.95	39.62	22.42	5.29	2.76	91.95	8.38	37.86	53.76
Orchard	37.20	45.07	17.74	5.17	3.11	91.71	3.64	40.38	55.97

**Table 4:** Each variable’s contribution rate and importance for estimating the CES flow in different land use types.

Variable	Contribution (%)				Importance (%)			
	Paddy Field	Dry Farmland	Tea Garden	Orchard	Paddy Field	Dry Farmland	Tea Garden	Orchard
Shape	12.5	3.1	21.5	1.3	12.1	4.1	2.2	1.9
Contiguity	5.5	62.2	0.5	21.1	8.3	54.5	0.3	16.5
Landscape diversity	5.5	7.0	7.0	2.9	8.7	7.6	13.9	2.9
Distance to water	1.1	6.9	9.1	34.3	1.2	8.8	10.2	21.2
TRI	23.9	4.4	43.5	2.1	30.5	12.4	51.4	4.6
Accessibility	51.6	16.6	18.4	38.3	39.2	12.6	22.0	53.0
